# Apelin-13 and Asprosin in Adolescents with Anorexia Nervosa and Their Association with Psychometric and Metabolic Variables

**DOI:** 10.1192/j.eurpsy.2023.289

**Published:** 2023-07-19

**Authors:** K. Jowik, M. Dmitrzak-Węglarz, N. Pytlińska, A. Jasińska-Mikołajczak, A. Słopień, M. Tyszkiewicz-Nwafor

**Affiliations:** 1 Department of Child and Adolescent Psychiatry; 2 Department of Psychiatric Genetics; 3 Department of Adult Psychiatry, Poznan University of Medical Sciences, Poznań, Poland

## Abstract

**Introduction:**

Anorexia nervosa (AN) is a widespread, metabo-psychiatric disorder with high relapse rates, comorbidity, and mortality. Many regulatory proteins and neurohormones studied to date play essential roles in the etiopathogenesis of eating disorders and the maintenance of psychopathological symptoms. Nevertheless, the regulatory and pathophysiological mechanisms of AN are still poorly understood.

**Objectives:**

The present study aimed to investigate the plasma levels of asprosin (ASP) and apelin-13 (APE-13) in malnourished (AN1) and partially cured (AN2) adolescent patients with AN. Correlations between protein levels and several dimensions of AN symptomatology, such as eating disorder, depressive, and obsessive compulsive symptoms, were investigated.

**Methods:**

Sixty-four patients aged 11–18 years admitted to the Department of Child and Adolescent Psychiatry in the acute phase of AN participated in the study. Between the 1st and 3rd days of admission, patients with AN (AN1) underwent psychometric evaluation, height and weight assessment, and 15 mL of blood was drawn. The same procedures were repeated at a second time point about 11.2 ± 2.3 weeks later, after partial normalization of body weight on the day of discharge (AN2). The control group (CG) normal-weight girls with no history of psychiatric disorders, recruited from among the students of a local school. The Eating Attitudes Test (EAT-26), Beck Depression Inventory (BDI) Hamilton Depression Scale (HAMD) and Yale–Brown Obsessive Compulsive Scale (CYBOCS), were used to assess eating disorder symptoms, depression, obsessions and compulsions. Patients were included in a nutritional rehabilitation program. Daily caloric intake was 2000–2500 kcal and was gradually increased to 3500–4000 kcal depending on weight gain (1.0–1.5 kg per week).

**Results:**

APE-13 levels were higher in the AN1 group than in the post-realimentation and the CG group. APE-13 levels were independent of insulin and glucose levels. Plasma ASP levels increased with increasing body weight in patients with AN, correlating with the severity of eating disorder symptoms in emaciation.

**Image:**

**Figure:**
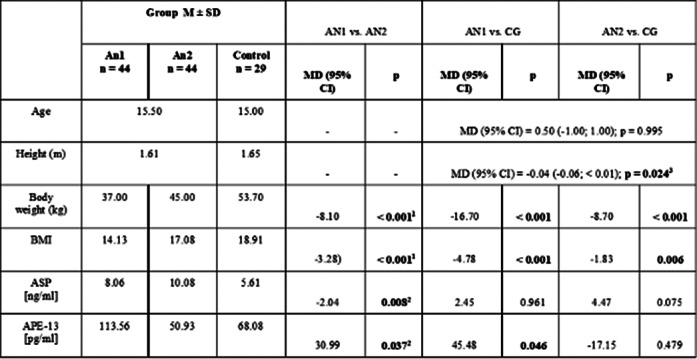

**Image 2:**

**Figure:**
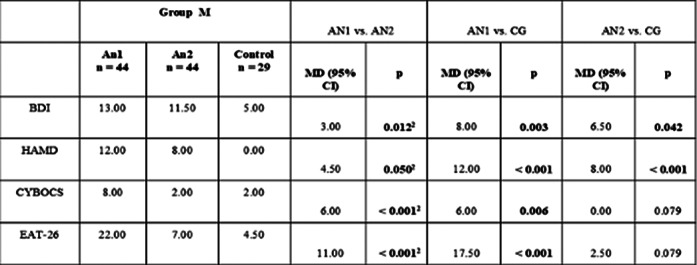

**Image 3:**

**Figure:**
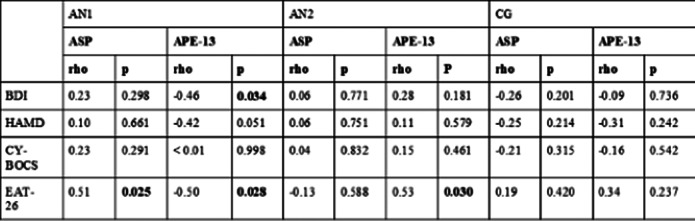

**Conclusions:**

The presented data suggest that APE-13 and ASP may be AN’s biomarkers-regulation of eating behavior by APE-13 and ASP, the close relationship between them and emotional behavior, and changes in neurohormone levels in patients with eating and affective disorders seem to support these hypotheses. Moreover, their plasma levels seem to be related to the severity of psychopathological symptoms of eating disorders.

**Disclosure of Interest:**

None Declared

